# Treatment of Infantile Hemangioma in Regional Hospitals With eHealth Support: Evaluation of Feasibility and Acceptance by Parents and Doctors

**DOI:** 10.2196/resprot.3418

**Published:** 2014-11-03

**Authors:** Marlies de Graaf, Joan EE Totté, Harmieke van Os-Medendorp, Wilco van Renselaar, Corstiaan C Breugem, Suzanne GMA Pasmans

**Affiliations:** ^1^Wilhelmina Children’s Hospital, University Medical Center UtrechtDepartment of Pediatric Dermatology and AllergologyUtrechtNetherlands; ^2^University Medical Center UtrechtDepartment of Dermatology and AllergologyUtrechtNetherlands; ^3^Erasmus MC University Medical Center RotterdamDepartment of DermatologyRotterdamNetherlands; ^4^Patient 1 BVAlmereNetherlands; ^5^Wilhelmina Children’s Hospital, University Medical Center UtrechtDepartment of Pediatric Plastic SurgeryUtrechtNetherlands

**Keywords:** eHealth, personal health record, e-consulation, tertiary teledermatology, Internet, acceptance, usability, infantile hemangioma, child

## Abstract

**Background:**

Since beta blockers became the preferred treatment for infantile hemangiomas (IH), the number of patients eligible for treatment is increasing. Currently treatment of IH with beta blockers is mainly reserved for expert centers, where wait times are lengthening. This demonstrated the need for development of a more efficient and accessible way of providing care for children needing treatment for IH. An eHealth intervention, Hemangioma Treatment Plan (HTP), was developed to treat IH in regional hospitals with online support from an academic doctor.

**Objective:**

Our goal was to evaluate the feasibility of the eHealth intervention by determining its use, acceptance, and usability. By evaluating the feasibility, usage can be predicted and points for improvement can be defined, thereby facilitating implementation of the intervention.

**Methods:**

Parents of children with an IH, presenting between October 2012 and November 2013 at the tertiary expert Center for Congenital Vascular Anomalies Utrecht, requiring treatment with a beta blocker, were asked to participate in the digital HTP. Both parents and regional doctors were sent a study questionnaire. Acceptance and usability of the HTP were evaluated by using the modified Technology Acceptance Model.

**Results:**

A total of 31 parents and 22 regional doctors participated in the eHealth intervention and received the questionnaire, and 25 parents and 15 doctors responded (response rates respectively 81% and 68%). A majority of the parents (96%, 24/25) and the regional doctors (87%, 13/15) considered the eHealth intervention useful in the care for IH. Most parents (76%, 19/25) and over half of the regional doctors (53%, 8/15) found the HTP easy to use. Technical problems using the HTP were reported by 28% (7/25) of the parents and 73% (11/15) of the doctors. The majority of parents (92%, 23/25) felt positive about usage of the HTP during treatment of their child. All regional doctors (100%, 15/15) felt positive about transition of treatment from the tertiary expert center to them, and 93% (14/15) felt positive about using the HTP.

**Conclusions:**

Our eHealth intervention shows good feasibility, especially among parents. Improvement with respect to technical problems, training of regional doctors, and achieving organizational support might be needed for successful implementation in the future.

## Introduction

Infantile hemangiomas (IH) are common benign vascular tumors found in approximately 4-10% of Caucasian infants [1,2]. Most IH have an uncomplicated course, and a general “wait and see” policy is often justified [3]. However, 24% of patients with IH experience complications, like ulceration, bleeding, functional impairment, life-threatening risk, or cosmetic risk, of which 38% need treatment [3]. In 2008, the efficacy of propranolol, a non-selective beta blocker, in the treatment of complicated IH was discovered, and propranolol became the primary treatment of choice [4,5]. Atenolol, a selective beta-blocker, has also been described as effective in the treatment of IH, showing less severe side effects compared to propranolol [6]. Since beta blocker treatment for IH shows less adverse effects and is less invasive compared to previously used treatment options (like systemic corticosteroids, interferon, and vincristine), the number of patients eligible for treatment is increasing [5-7].

Treatment of IH is currently taking place particularly with multidisciplinary expert teams in tertiary academic centers, like the Center for Congenital Vascular Anomalies Utrecht (CAVU), Wilhelmina Children’s Hospital, University Medical Center Utrecht, the Netherlands. However, our wait times are lengthening due to the increasing patient flow. This, together with the sometimes long travel distances for parents, shows the need for development of a more efficient and accessible way of providing care for children needing treatment for IH.

eHealth can be defined as the use of all information and communication technology to improve health and health care [8]. In the Netherlands, there is an eHealth intervention to help parents in the diagnostic process of the vascular skin lesion of their child called Aardbeivlek [9]. This eHealth intervention helps parents correctly diagnose and evaluate an IH after completing an eLearning module. To improve health care in children with skin diseases, we have developed the online pediatric Skin House (huidhuis.nl), a digital interactive platform for information, treatment, and exchange of expertise of pediatric skin diseases, which is accessible to patients, their parents, and health care providers. This study describes a part of the pediatric Skin House: a Web-based personalized eHealth intervention called Hemangioma Treatment Plan (HTP) for treating IH. This eHealth intervention consists of a digital interactive platform of information, treatment, and expertise about IH. It also includes a personal health record (PHR) owned by the patient who gives access to the professionals involved. The aim of this eHealth intervention is efficient and easily accessible care for children with IH by making disease knowledge, treatment protocols, and the PHR easily available to both parents and health care providers. By using the eHealth intervention, children with IH can be treated by their medical doctor in a regional hospital with online support of the experts of the CAVU team (tertiary academic care).

The goal of this study was to evaluate the feasibility of this eHealth intervention by determining its use, acceptance, and usability by parents and doctors. By evaluating the feasibility, usage can be predicted [10,11] and points for improvement can be defined, thereby facilitating implementation of the intervention in the future.

## Methods

### Design

A cross-sectional study was performed to evaluate the feasibility of the eHealth intervention judged by parents and medical doctors.

### Participants

#### Parents

Parents of children with an IH, presenting between October 2012 and November 2013 at the CAVU and requiring treatment with an oral beta blocker, were asked to participate in the digital HTP. Indications for treatment were (risk of) ulceration, (risk of) functional damage, and (risk of) cosmetic damage. Parents who did not have access to a computer were excluded. Other exclusion criteria were no/insufficient knowledge of the Dutch language and complications of the IH requiring specialized multidisciplinary care. Decisions on inclusion and exclusion based on above mentioned criteria were made by an expert member of the CAVU team.

#### Medical Doctors

Regional medical doctors (pediatricians and dermatologists) were informed about the HTP by a digital mailing and/or by personal invitation. After showing their interest in participation, they were included in our database. Children who needed beta blocker treatment were referred to the regional medical doctor closest to their home/residence. Other inclusion criteria were access to Internet and ability to measure blood pressure (BP) in a young child.

The study was approved by the ethics committee of the University Medical Center Utrecht.

### Intervention

#### Hemangioma Treatment Plan

In order to achieve more efficient and easily accessible care for IH, an eHealth intervention was designed (in Dutch), called HTP. This interactive digital treatment platform consisted of multiple elements providing the following functions: (1) storage and sharing of patient health information (in a digital PHR) through a secured Web-based portal, (2) providing information about the disease and treatment protocols, (3) facilitating communication between parent and the medical doctor (e-consult), and (4) facilitating communication between the regional and academic doctor (tertiary teledermatology).

The purpose of the HTP is first to warrant safe transition of IH treatment from academic doctors to regional doctors by using digital support. Second, its purpose is to involve parents in the care for IH by making information and contact with doctors more efficient and accessible.

The HTP, including a PHR, was developed in collaboration with Patient1, a private company that offers a secured digital health platform. The health platform includes a PHR, professional health care records, digital research center, and information websites like pediatric Skin House. The PHR was compliant with all Dutch rules and regulations with respect to privacy protection and was checked and approved by the Dutch Privacy Protection Authority. Participation in the HTP was free of charge.

Instructions on the use of the HTP were given to the parents verbally and in writing. Parents created an individual account for their child on the PHR website by registering name, birth date, and personal identification number of the child. By using a password, safe uploading of personal information on the website was guaranteed. The PHR account contained information about IH treatment, the HTP, a message-function (for e-consultation and tertiary teledermatology), and a facility to upload photographs and record effect and side effects. Parents created the PHR themselves and gave the medical doctors access to the PHR.

Regional doctors were given instructions on the use of the HTP in writing and sometimes verbally by phone. Regional doctors also registered at Patient1 using name, birth date, and a personal identification code for health care providers in the Netherlands). After verification and authorization by Patient1 and the parents, the regional doctor involved had access to the individual account of the child being treated.

#### Treatment Protocol

Treatment of the IH was started with atenolol at the tertiary center, after evaluation of possible contra-indications (which included an electrocardiogram) [12]. Follow-up was performed by the regional doctor near the patient’s home, using the HTP. At the age of 1 year, all children were seen by the CAVU team of the academic center to decide whether or not to stop treatment. Regional doctors used an IH treatment protocol for follow-up of the child treated for IH, accessible via the HTP. The protocol describes set moments for consultation and instructions on how to monitor effects and side effects of treatment. Prior to each consultation, parents uploaded photographs of the IH of their child, scored the severity of the IH, and completed standardized questionnaires on potential side effects. With the information provided by the parents and findings during the consultation, the regional doctor decided on further treatment policy, guided by the IH treatment protocol. Findings and policy were reported in the PHR of the HTP.

**Figure 1:**
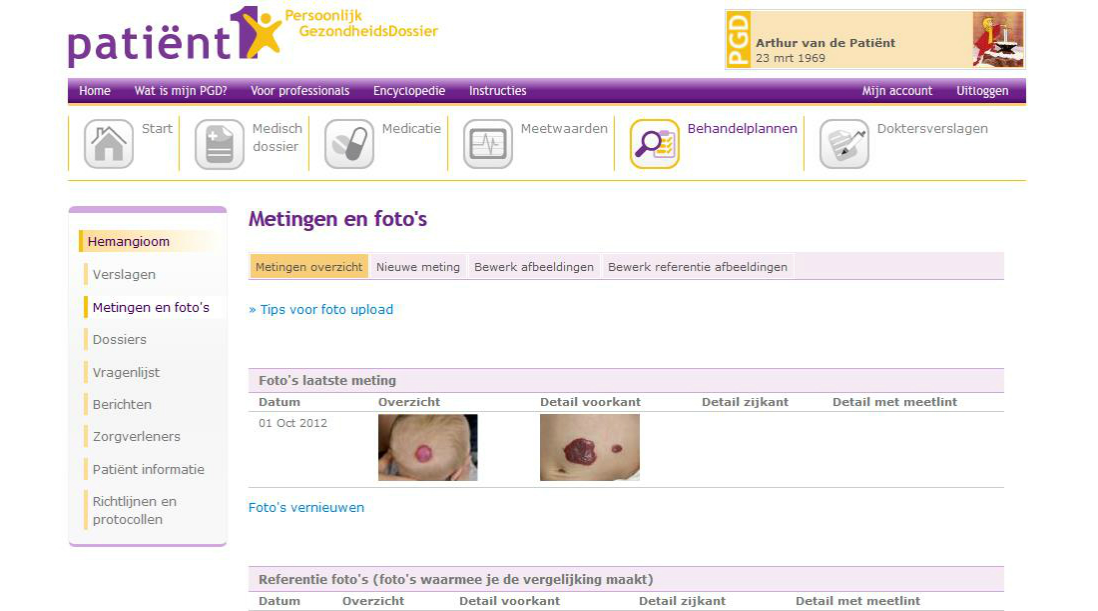
Screenshot of the Hemangioma Treatment Plan (in Dutch) showing the page where parents upload a photo of the hemangioma prior to consultation (fictitious patient in a test environment).

**Figure 2:**
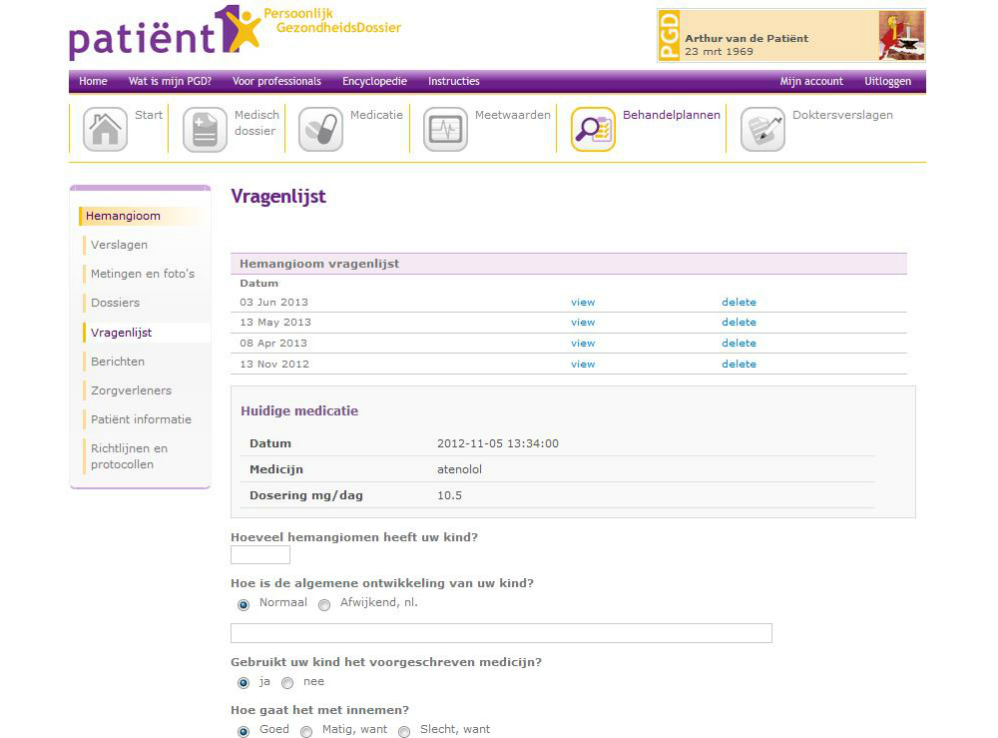
Screenshot of the Hemangioma Treatment Plan (in Dutch) showing the page where parents fill in standardized questionnaires about side-effects prior to consultation (fictitious patient in a test environment).

When advice from the academic doctor (expert dermatologist of the CAVU team) was required, the parent or the regional doctor could send a message via the HTP (respectively e-consult or tertiary teledermatology). These questions were answered within 3 working days by the academic doctor. For urgent situations, such as severe side effects of treatment, parents were instructed to contact the academic doctor who was available 24 hours a day by phone. Patients and doctors received automatic notification messages in their personal email inbox when a message was placed in the HTP. Figures 1 and 2 show screenshots of the HTP.

### Variables and Measurement

A questionnaire to evaluate feasibility was developed consisting of the variable use, acceptance, and usability of the eHealth intervention.

The questionnaire was developed based on a modified Technology Acceptance Model (TAM). The TAM model, proposed by Davis in 1989, is based on the Theory of Reasoned Reaction and proposes that Perceived Ease of Use (PEU) and Perceived Usefulness (PU) predict user acceptance of information technology [10,11,13]. Technology acceptance is defined as “an individual’s psychological state with regard to his or her voluntary or intended use of a particular technology” [14]. The TAM has been tested for the prediction of adoption of telemedicine by health care professionals and can predict technology acceptance in both obligatory and voluntary usage settings [15,16]. It is suitable for both genders, various age groups, most cultures, and for individuals of all levels of information technology competency [16]. To determine the use, acceptance, and usability of our eHealth intervention, we modified the TAM by adding the dimension “attitude towards use” to the original TAM. Attitude can be defined as “the perception by an individual of the positive or negative consequences related to adopting the technology”. Behavioral intention is also determined by attitude, which is influenced by PU and PEU [17]. Questions to evaluate use, acceptance, and usability were developed following the modified TAM.

After at least one consultation with the regional doctor, both parents and doctors were sent a structured study questionnaire. The study questionnaire of the parents and doctors consisted of 38 and 29 questions respectively, grouped into 3 variables (demographic information, use, acceptance and usability) (Table 1). Acceptance and usability were subdivided by the three dimensions of the TAM (PU, PEU, and attitude). On a 3-point scale (agree-no agreement/no disagreement-disagree), we rated 22 questions for the parents and 15 for the doctor. Six and nine questions respectively of the parent and doctor questionnaire could be answered with yes or no, and with the final question, parents and doctors were asked to rate the eHealth intervention on a 0-10 scale (0=very bad, 10=excellent). Apart from the generic questions, all questions contained room to clarify the answer. At the end of the questionnaire, there was an open field for comments and suggestions. Prior to the study, it was determined that an average of 90% of the parents and doctors had to score the items positively regarding feasibility to qualify the eHealth intervention as feasible.

**Table 1:** Questions used to evaluate feasibility of the eHealth intervention.

Variable	Dimension	Related questions		Example
		Parent questionnaire	Doctor questionnaire	
**Demographic information**				
		1-6, 9, 11, 13	1-3, 6	Parent: gender, age, education level, residence, treatment indication
				Doctor: gender, age, medical specialism
**Use**				
		7e-f, 10, 16, 17h	4c-d, 8, 10	Treatment at the regional doctor corresponds with information given by academic center
**Acceptance and usability**				
	Perceived usefulness	8, 17e, 19, 20a-c	4e, 5, 9f	The e-consult function of the HTP is useful
	Perceived ease of use	15, 17d, 17f-g, 17i-j,	7, 9a-e, 9g-h, 12, 13a-d	The instruction letter of the HTP is understandable and clear
	Attitude	7a-d, 7g, 12, 14, 17a-c, 18, 21	4a-b, 9i, 11, 14	I feel positive about treatment by a regional doctor, with digital support from an expert

### Statistics and Analyses

User statistics were recorded. The number of e-consultations, tertiary teledermatology consultations, and responding times were calculated.

Descriptive analyses were used to evaluate the use, acceptance, and usability.

## Results

### Overview

A total of 31 parents and 22 regional doctors participated in the HTP and received the questionnaire; 25 parents and 15 regional doctors responded (response rate of respectively 81% and 68%). Reasons for not responding on the questionnaire are unknown. Parent and regional doctor characteristics are shown in Table 2.

At the start of treatment, all children were ≤5 months of age. All parents and doctors proved themselves experienced with use of the computer and Internet. Each regional doctor treated an average of 1.5 patients (range 1-4). The mean distance from parents’ residence to the tertiary expert center was 52 kilometers.

**Table 2:** Characteristics of the parents and regional doctors.

Characteristics		Parents, n=25	Regional doctors, n=15
Male-female ratio		1:7.3	1:1.5
**Age of respondent, years**			
	Mean (SD)	32.1 (4.1)	44.9 (9.4)
	Median (range)	32.0 (25-41)	39.0 (36-62)
**Medical specialty, n/N (%)**			
	Dermatologist		7/15 (47)
	Pediatrician		8/15 (53)
**Level of education, n/N (%)**			
	Low	0/25 (0)	
	Moderate	6/25 (24)	
	High	18/25 (72)	
	Unknown	1/25 (4)	
**Indication for treatment, n/N (%)** ^a^			
	Cosmetic	9/25 (36)	
	Functional	14/25 (56)	
	Ulceration	5/25 (20)	

^a^In some cases there were multiple indications for treatment.

### Use

All parents and doctors (regional and academic doctors) used the HTP. The e-consult function of the HTP was used by all parents. The average number of e-consultations was 0.5 per parent/month. E-consultations were mostly sent to (and answered by) the academic doctor, 106/112 (95%) messages, within an average time of 2 days. All parents found that their e-consultations were answered adequately.

Eight regional doctors contacted the academic doctor. Of the 8 regional doctors, 7 (88%) found the academic doctor easily accessible for consultation and questions; 40% (6/15) of the regional doctors used tertiary teledermatology to contact the academic doctor. Others contacted the academic doctor by email or phone. Four of the 15 regional doctors (27%) used the e-consult function to communicate with parents. Some contact moments between doctors and patients were not reported in the HTP, whereas doctors did report in the hospital’s own electronic patient dossier.

The different functions of the HTP (e-consult, uploading photographs, completing questionnaires) were used by 19/25 (76%, range 32-100%) of the parents.

Many of the parents (63%, range 52-76%) agreed that treatment by the regional doctor corresponded with the information given by academic center. Parents saved time and costs because of treatment at a regional doctor in respectively 58% and 48% of the cases.

Of the regional doctors, 93% (14/15) felt informed enough to treat patients with IH.

### Acceptance and Usability

#### Perceived Usefulness

A majority of the parents (24/25, 96%) and regional doctors (13/15, 87%) considered the eHealth intervention useful in the care for IH. Parents agreed that different functions (e-consult, PHR, etc) of the HTP were useful (average 21.5/25, 86%, range 84-88%). The different functions of the HTP (eg, IH treatment protocol, access to information about side effects and photographs prior to consultation) were useful according to an average of 87% (range 80-93%) of the regional doctors. There were 7/13 (54%) regional doctors and 22/25 (88%) parents that thought the e-consult function to contact each other was useful. The tertiary teledermatology was thought to be useful by many of the regional doctors who used tertiary teledermatology (75%, 9/12).

#### Perceived Ease of Use

Instructions on the HTP and patient information were clear according to 92% (23/25) and 96% (24/25) of the parents respectively. Instructions on the HTP and the IH treatment protocol were clear according to 73% (11/15) and 80% (12/15) of the regional doctors respectively. Many of the parents (76%, 19/25) and just over half (53%, 8/15) of the regional doctors agreed on the statement that the HTP is easy to use. Technical problems using the HTP were reported by 28% (7/25) of the parents and 73% (11/15) of the doctors.

#### Attitude

Most of the parents (88%, 22/25) felt positive about treatment at a regional doctor, and many parents (68%, 17/25) found that treatment at a regional doctor felt safe. All 15 regional doctors (100%) felt positive about transition of treatment from the tertiary expert center to them, and 92% (23/25) of the parents and 93% (14/15) of the regional doctors felt positive about usage of the HTP. Almost all parents (96%, 24/25) found the HTP was worth the time investment. Although, with 47% agreement, regional doctors reported that they have difficulties with the time investment in the HTP (documenting in the PHR and answering e-consultations). Finally, 72% (18/25) of parents felt more involved in treatment due to the HTP.

The average satisfaction rates parents and regional doctors gave the eHealth intervention on a 0-10 scale were 7.7 (SD 0.75).and 7.3 (SD 1.4), respectively. An overview of the main results is given in Table 3.

Comments and suggestions were evaluated. Positive comments of the parents were given about improvement of access to health care professionals and saving time. Positive comments of the regional doctors included improvement of contact between parents and experts. Points of attention of parents were privacy issues and lack of trust in expertise of regional doctors. Regional doctors were concerned about time investment.

**Table 3:** Overview of the feasibility of the eHealth intervention sorted by variable.

Feasibility	Parents	n/N (%)	Regional doctors	n/N (%)
Use	Use of different functions (e-consult, uploading photographs, questionnaires) (mean)	19/25 (76) range 32-100	Use of different functions (tertiary teledermatology)	6/15 (40)
	E-consults were adequately answered	25/25 (100)	Informed enough about IH care	14/15 (93)
	Treatment at regional doctor corresponds with information given by academic center (mean)	15.7/25 (63) range 52-76	Easy contact with tertiary caretaker	7/8 (88)
Perceived usefulness	HTP is useful	24/25 (96)	HTP is useful	13/15 (87)
	Usefulness of different functions	21.5/25 (86) range 84-88	Usefulness of different functions (mean)	12.7/15 and 13/14 (87, range 80-93)
	Usefulness of e-consult	22/25 (88)	Usefulness of tertiary teledermatology	9/12 (75)
			Usefulness of e-consultation	7/13 (54)
Perceived ease of use	Instructions of the HTP	23.5/25 (94) range 92-96	Instructions of the HTP	11.5/15 (77) range 73-80
	The HTP is easy to use	19/25 (76)	The HTP is easy to use	8/15 (53)
	Technical problems	7/25 (28)	Technical problems	11/15 (73)
Attitude	Positive about treatment at secondary caretaker	22/25 (88)	Positive about treatment at secondary caretaker	15/15 (100)
	Positive about usage of HTP	23/25 (92)	Positive about usage of HTP	14/15 (93)
	Worth the time investment	24/25 (96)	Worth the time investment	7/15 (47)
	More involved in care	18/25 (72)		

## Discussion

### Principal Findings

This study describes an eHealth intervention to make the care for children with IH efficient and easily accessible using an online hemangioma treatment plan. Treatment of IH took place with regional doctors, supported by an expert at distance (academic doctor). The HTP was used to facilitate transition of treatment to regional doctors and to involve parents in the care for IH. Evaluation of the feasibility of this new way of providing care was performed by studying the small group of first patients and regional doctors who participated in this newly developed eHealth intervention.

The feasibility according to the parents ranged from 63-100% and according to the regional doctors from 47-100% (Table 3). The predetermined percentage of 90%, necessary to qualify the eHealth intervention as feasible, was not always reached. However, almost all parents thought the HTP was useful and all regional doctors had a positive attitude towards the HTP.

Although most results on feasibility are positive, only 53% of the regional doctors found the HTP easy to use. This could be influenced by the fact that 73% of them experienced technical problems and that they were mostly instructed in writing. Most technical problems experienced by both parents and doctors related to logging in and uploading of photographs. The problems were most likely caused by prematurity of the technology itself. Problems of logging in are resolved now. The problem with uploading photographs was due to low capacity of the website and will be resolved in the near future. Technical problems might also be caused by a lack of adequate computer skills to use eHealth systems. Health care providers are the key driving force in pushing eHealth initiatives [18]. IT support (verbal) might facilitate eHealth acceptance and use [19].

Furthermore, the tertiary teledermatology to consult an academic doctor of the CAVU team was seldom used by the regional doctors. Mostly they consulted the CAVU team by phone or by sending emails. Probably the regional doctors were not used to this tool for consulting a colleague, and the fact that parents could see the content of the questions of the regional doctor may have contributed. However, studies have shown that tertiary teledermatology might improve communication between regional and academic doctors and might reduce wait times [20,21]. They have advantages over telephone consultations, which require the need for both parties to be available at the same time, and email, which does not meet current privacy requirements for sharing personal health information [20,22].

Secondly, regional doctors were not positive about the e-consult with the parents. Only about half (54%, 7/13) of regional doctors considered the e-consultation useful. A possible explanation is that these doctors might expect e-consultations to be time consuming (74% of the regional doctors found the HTP worth the time investment) or that they are not used to working with e-consultations. However, it has been shown that the use of e-consultations in dermatology is feasible and the majority of e-consultations take less than 10 minutes for the medical doctor to answer [20,23,24]. It has been shown that e-consultations improve access to specialty care [25]. On the other hand, Palen et al [26] found that having online access to medical records and clinicians was associated with increased use of clinical services. However, this has been debated by others [27,28]. Further research should point out the consequences of e-consultations for time investment and usual care.

Some parents felt that the regional doctor was less experienced with IH care. However, in our opinion, this is probably only a temporary problem since studies have demonstrated that online health communities of doctors from different echelons and patients can be used to exchange medical experience and knowledge and that knowledge of participants increased and the adherence to guideline recommendations improved [29,30]. Partial transition of treatment of IH to secondary centers combined with support by an expert might have an educational value for the regional doctors. This is confirmed by the fact that 87% of the regional doctors agreed that the HTP was of educational value for them. In the long term, increased knowledge about IH treatment and treatment indications might result in better recognition and treatment of children with IH at risk for complications. In the short term, increasing the knowledge about treatment of IH among regional doctors (eg, through eLearning courses) might make them and the parents more comfortable with treatment in regional centers.

Some of the parents included in this study mentioned privacy issues. Security and privacy issues are consistently found in studies as influencing patients’ interest in digital PHRs [31]. Adequate verbal instruction to parents about (the prematurity of) the intervention and about security is important in further implementation. The implementation of the HTP requires a different way of acting and thinking from both doctor and patient. Satisfaction of eHealth interventions has rarely been studied [32]. High patient satisfaction was also seen in a Dutch eHealth intervention that includes an eczema portal combining e-consulting, monitoring, and self-management training for patients and parents of young children with atopic dermatitis [33,34]. Overall parents were positive about the eHealth intervention. From the perspective of the regional doctor, feasibility of treatment at local hospitals and system usability of the HTP should be further adapted to their needs to enhance acceptation, actual usage of the HTP, and implementation on a larger scale. It is known that medical doctors have had some reservations about moving forward in the area of eHealth and PHRs, partly because of concern that they will be bombarded with questions and that patients will have trouble interpreting their findings [35,36]. However, most of the empirical experiences suggest that these problems do not represent major issues when patients are provided with and adopt PHRs [37,38]. Solutions to reduce time investment could involve specialized nurses in the triage of e-consultations and creating a link between the patient file of the hospital and the PHR of the eHealth intervention. Nevertheless, results of this early evaluation of the HTP should be interpreted with consideration of the psychology that goes along with changes in management. Hands-on training for the set-up could be necessary for structural implementation of the e-consultation functionality [25]. Besides patient-doctor interaction, workforce items such as workload and workflow, as well as contextual factors like institutional policy regarding eHealth, influence the implementation of eHealth interventions [39]. To realize treatment of IH on a larger basis at local hospitals, clear referral policy should be made. Regional doctors should agree on how to facilitate treatment of IH on a larger scale. Academic doctors have an important role in assuring the quality of care as they are expected to recognize those patients that require treatment at the tertiary center. Hospital management of tertiary centers should incorporate e-consulting in daily practice to ensure the academic doctor can meet this important role.

### Limitations

The results of our study must be interpreted with caution given the small sample size of both parents and doctors and the prematurity of the intervention. Furthermore, parents had a relatively high education level and were therefore not representative of the general population.

The implementation of eHealth interventions will incur costs. However, the transition of IH treatment to secondary centers might save (in)direct health care costs. Health insurance does already reimburse the implementation of eHealth in some fields of medicine. However, there is still no funding for the care provided through the eHealth interventions. Studies have shown that eHealth, combining e-consultations, monitoring, and self-management training, could lead to cost-savings, and e-consultations could reduce the number of face-to-face consultations [20,23,24,40]. In this study, parents reported a time and cost reduction with respect to traveling due to the use of the HTP. Lower costs can be expected due to a lower number of face-to-face consultations at the tertiary academic center. There might even be situations where digital contact through the HTP replaces face-to-face contact. Wait times will shorten, and more new patients can be seen in a shorter time. Lower indirect costs can be expected due to lower work-absenteeism, as care can be received closer to home. Further studies are necessary to confirm this.

### Conclusions

The HTP is a new care innovation that was and will be continuously improved according to user feedback. Points for improvement are resolving the technical problems, such as extending the capacity for uploading photographs, providing more detailed training for regional doctors, and taking care of organizational support. The HTP was a pilot to evaluate the feasibility of treating patients in a regional hospital with online support of the academic center and was part of the pediatric Skin House. After some improvement, this eHealth intervention could be a helpful tool for efficient and accessible care for IH and might be used to increase cooperation between different sectors of health care (primary, secondary, and tertiary care).

Our eHealth intervention to improve the efficiency and accessibility of care for children with IH shows good feasibility, especially among parents. Improvement with respect to technical problems, training of regional doctors, and achieving organizational support might be needed for successful implementation in the future.

## Abbreviations

CAVU: Center for Congenital Vascular Anomalies Utrecht
HTP: hemangioma treatment plan
IH: infantile hemangioma
PEU: perceived ease of use
PHR: personal health record
PU: perceived usefulness
TAM: Technology Acceptance Model
